# Pre-extubation functional residual capacity and risk of extubation failure among patients with hypoxemic respiratory failure

**DOI:** 10.1038/s41598-020-58008-4

**Published:** 2020-01-22

**Authors:** Hui-Chuan Chen, Sheng-Yuan Ruan, Chun-Ta Huang, Pei-Yu Huang, Jung-Yien Chien, Lu-Cheng Kuo, Ping-Hung Kuo, Huey-Dong Wu

**Affiliations:** 1 Division of Respiratory Therapy, Department of Integrated Diagnostic and Therapeutics, National Taiwan University Hospital, Taipei, Taiwan; 2 Division of Pulmonary and Critical Care Medicine, Department of Internal Medicine, National Taiwan University Hospital and College of Medicine, Taipei, Taiwan

**Keywords:** Outcomes research, Risk factors

## Abstract

Hypoxemic respiratory failure is usually accompanied with a certain extent of consolidation and alveolar derecruitment, which may still be present even after the patients have achieved the status of readiness to extubate. Functional residual capacity (FRC) is an indicator of lung aeration. This study aimed to evaluate whether pre-extubation FRC is associated with the risk of extubation failure in patients with hypoxemic respiratory failure. We prospectively included 92 patients intubated for hypoxemic respiratory failure. We used a technique based on a nitrogen multiple breath washout method to measure FRC before the planned extubation. The median FRC before extubation was 25 mL/kg (Interquartile range, 20–32 mL/Kg) per predicted body weight (pBW). After extubation, 20 patients (21.7%) were reintubated within 48 hours. The median FRC was higher in the extubation success group than in the extubation failure group (27 versus 21 mL/Kg, p < 0.001). Reduced FRC was associated with higher risk of extubation failure (odds ratio, 1.14 per each decreased of 1 mL/Kg of FRC/pBW, 95% CI, 1.05–1.23, p = 0.002). In conclusion, pre-extubation FRC is associated with the risk of extubation failure. Reduced FRC may be incorporated into the traditional risk factors to identify patients at high risk for extubation failure.

## Introduction

Hypoxemic respiratory failure is the leading cause for commencing invasive mechanical ventilation in the intensive care unit (ICU). The common causes of hypoxemic respiratory failure include pneumonia, lung edema and atelectasis^1^. The pathophysiology of hypoxemia in these conditions is related to the loss of alveolar space due to consolidation and alveolar derecruitment. Nevertheless, the affected lungs might not be fully aerated after the patients survive the acute critical illness. A certain extent of alveolar space loss may remain, even after patients have recovered to the status of readiness to extubate. Functional residual capacity (FRC) is a measure of lung function that can be used to quantify the extent of loss of alveolar space. Reduced FRC before extubation may have negative impact on subsequent weaning outcome^2^. However, whether FRC is associated with the risk of extubation failure has not been evaluated.

FRC or end-expiratory lung volume is a measure of the lung volume during breathing at the average end-expiratory level^3^. Previous studies suggested that FRC was a good indicator of aeration and recruitment of lung tissue^4,5^. The normal FRC in healthy individuals is approximate 30–35 mL/Kg per predicted body weight in the sitting position^6–8^. Traditionally, FRC can be measured by means of gas dilution, nitrogen washout, body plethysmography or computed tomography^2,3,9^. These measurement techniques require standardized pulmonary function equipment, which is difficult to use in ventilated patients in the ICU. However, new techniques are now available to measure FRC in ventilated patients without interrupting mechanical ventilation^10,11^. Based on a nitrogen multiple breath washout technique, a reliable FRC measuring method has been integrated into ventilators and can be easily used in the ICU^10^.

In this study, we hypothesized that the extent of consolidation and alveolar derecruitment in patients recovering from hypoxemic respiratory failure could be quantified with FRC measurement and that reduced FRC has negative impact on extubation outcome. We aimed to evaluate the association between pre-extubation FRC and risk of extubation failure among patients with hypoxemic respiratory failure.

## Methods

### Study design and patients

This prospective cohort study was conducted in a medical ICU in a university-affiliated teaching hospital in Taiwan from November 2016 through December 2017. This study aimed to test whether pre-extubation FRC was linked to extubation outcome in patients intubated for hypoxemic respiratory failure. Adult patients receiving mechanical ventilation in the ICU were screened for eligibility during the study period. The eligibility criteria were (1) intubation for hypoxemic respiratory failure, (2) use of mechanical ventilation for at least two days, (3) passing a spontaneous breathing trial (SBT) and (4) being prepared to be extubated by primary care physicians. The exclusion criteria were intubation for hypercapnic respiratory failure, requiring an artificial airway for medical reasons, having a do-not-resuscitation order and refusal to reintubate after extubation.

### Ethical approval and informed consent

The study was performed in accordance with current ethical guidelines (Declaration of Helsinki) and was approved by the Research Ethics Committee of National Taiwan University Hospital (No.: 201606049RINB). Informed consent was obtained from each participant or the next of kin.

### Weaning protocol

All study subjects were put on Engstrom ventilators (GE Healthcare, Chicago, USA) during the study period. Each subject was screened daily for readiness to an SBT. The criteria of readiness for an SBT were FiO_2_ ≤ 0.4, positive end-expiratory pressure (PEEP) ≤ 8 cmH_2_O, no worsening of non-pulmonary organ systems and approval by the primary care physician to start daily SBT. Either a T-piece breathing trial or low-level pressure support was used as an SBT. The criteria for SBT success were determined according to previous guidelines^12^. The criteria for readiness to extubate included the presence of adequate consciousness, adequate cough power and passing an SBT. The cuff-leak test was performed in patients intubated for ≥ 5 days or patients who had a history of difficult intubation, upper airway narrowing and traumatic intubation. After the patient passed an SBT, the decision to extubate was at the discretion of the primary care team. Prophylactic noninvasive ventilation (NIV) support after extubation is not a routine practice in the hospital, except for patients with severe chronic obstructive pulmonary disease.

### Measurement of functional residual capacity

We used the FRC INview^TM^ tool (GE Healthcare, Chicago, USA) to measure FRC in this study. This technique is based on a nitrogen multiple breath washout method, which determines FRC by measuring the change in lung nitrogen volume after a change of at least 10% in the inspired oxygen fraction^10^.

We performed FRC measurement before study subjects were to be extubated. The subjects were put in a semi-recumbent position at 45° and ventilated with pressure support of 5 cm H_2_O and PEEP of 5 cm H_2_O. The cuff pressure of the endotracheal tube was checked to prevent air leakage and endotracheal suction was performed to remove possible airway secretions. The humidifier was temporally off during the measurement. After a steady state had been achieved for at least 10 minutes, FRC was measured using FRC INview^TM^. The mean value of two readings of FRC was recorded as the patient’s FRC. If the variation between the two FRC measurements exceeded 25%, the measurement process was repeated after another steady state of respiration had been achieved.

Because there are no established reference values of FRC for the Chinese population, we recorded the ratio of FRC to predicted body weight (FRC/pBW) in order to evaluate the change in FRC. Predicted body weight was calculated as 50 + 0.91 × (height [cm] – 152.4) for men, and as 45.5 + 0.91 × (height [cm] – 152.4) for women. The normal range of FRC/pBW in healthy individual is estimated to be 30–35 mL/kg^6,7^.

### Data collection and outcome measure

We recorded demographic data of the subjects, causes of respiratory failure, Acute Physiology and Chronic Health Evaluation II (APACHE II) scores, underlying comorbidities, ICU admission date, intubation and extubation date, and extubation outcome. Tidal volume, minute ventilation and rapid shallow breathing index (RSBI) were measured using a haloscale respirometer according to the standard care protocol of the study hospital.

The outcome of interest was extubation success, defined as remaining ventilator free and alive at 48 hours after extubation^13^. We compared FRC in patients with extubation success and extubation failure, and evaluated the discriminatory power of FRC for extubation outcome. The associations between FRC versus PaO_2_/FiO_2_ and FRC versus tidal volume of spontaneous breathing were evaluated as explanatory outcomes.

### Statistical analysis

We used logistic regression model to evaluate the association between FRC and extubation outcome. The sample size calculation was based on the assumption of the probability of extubation failure being 0.15 when the value of FRC/pBW was at the mean and the probability being 0.3 when the value of FRC/pBW was one standard deviation above the mean. A sample size of 90 patients was required to detect a significant difference between groups with a power of 80% and an α of 0.05.

Baseline characteristics of the study cohort were expressed as means with standard deviation (SD), medians with interquartile range (IQR) or number with proportion, as appropriate. We compared continuous variables with the Wilcoxon rank-sum test and proportions with the chi-square test. Pearson’s correlation coefficient was used to evaluate the linear correlation between FRC and variables of interest. Discriminatory power was determined by the area under the receiver operating characteristic curve (AUROC). We reported point estimates and corresponding 95% confidence intervals (95% CI).

We used Stata software version 15 (StataCorp, College Station, TX) for statistical analysis. Statistical tests were two-sided and a p-value of < 0.05 was considered to indicate statistical significance.

## Results

### Demographic and clinical characteristics

A total of 92 patients who were intubated for hypoxemic respiratory failure were enrolled. The characteristics of the study cohort are summarized in Table 1. The median age was 73 years (IQR, 61–81 years) and 44.6% were female. The leading causes of hypoxemic respiratory failure were pneumonia (62%), lung edema (8.7%) and acute respiratory distress syndrome (7.6%). On average, every patient had two sessions of SBTs (IQR, 1–3 times) before the planned extubation. The SBT method used for all the 92 patients was T-piece breathing. After extubation, 20 (21.7%) patients were reintubated within 48 hours. The comparison between extubation success and failure groups is shown in Table 1.

**Table 1:** Characteristics of the study cohort (n = 92).

Characteristics	Total cohort (n = 92)	Extubation outcome		
		Success (n = 72)	Failure (n = 20)	P value
Age, yr, median (IQR)	73 (61–81)	73 (60–81)	72 (64–83)	0.73
Female gender, n (%)	41 (44.6)	32 (44)	9 (45)	0.97
Height, cm, mean (SD)	160 (8)	160 (8)	161 (9)	0.77
Weight, kg, mean (SD)	57 (12)	56 (11)	62 (13)	0.04
APACHE II score, mean (SD)	23 (6)	22 (6)	26 (8)	0.02
Cause of hypoxemic respiratory failure, n (%)				
Pneumonia	57 (62.0)	46 (64)	11 (55)	0.67
Lung edema	8 (8.7)	6 (8)	2 (10)	
ARDS	7 (7.6)	6 (8)	1 (5)	
Lung cancer	6 (6.5)	5 (7)	1 (5)	
Pleural effusion or diseases	5 (5.4)	4 (6)	1 (5)	
Other	9 (9.8)	5 (7)	4 (20)	
Conditions before FRC measurement, mean (SD)				
RSBI, median (IQR)	66 (46–96)	63 (43–88)	94 (65–130)	0.01
PaO_2_/FiO_2_ ratio	337 (90)	349 (88)	291 (84)	0.01
PaCO_2_, mmHg	34 (7)	34 (6)	34 (8)	0.96
Minute ventilation, L/min	8.6 (3.6)	8.7 (3.5)	8.4 (4.0)	0.75
Tidal volume, mL	381 (168)	398 (169)	319 (150)	0.06
Maximal inspiratory pressure, cm H_2_O	37 (9)	37 (10)	36 (8)	0.51
Maximal expiratory pressure, cm H_2_O	36 (11)	36 (11)	35 (11)	0.70

APACHE, Acute Physiologic And Chronic Health Evaluation; ARDS, acute respiratory distress syndrome; FRC, functional residual capacity; IQR, interquartile range; RSBI, rapid shallow breathing index; SD, standard deviation.

### FRC and extubation outcome

**Figure 1:**
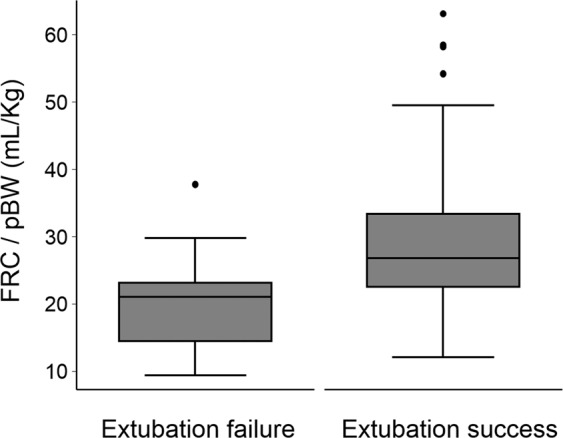
Box plots of functional residual capacity divided by predicted body weight (FRC/pBW) in extubation success and extubation failure groups (Wilcoxon rank-sum test, p < 0.001).

FRC was measured before extubation and the respiratory conditions before FRC measurement are summarized in Table 1. The median FRC was 1363 mL (IQR, 999–1729 mL) or 25 mL/kg (IQR, 20–32 mL/Kg) per pBW. Figure 1 shows the FRC/pBW of the extubation success and failure groups. The median FRC in the extubation success and failure groups was 27 mL/kg (IQR, 23–33 mL/Kg) and 21 mL/Kg (IQR, 14–23 mL/Kg), respectively. The difference reached statistical significance (p < 0.001).

**Figure 2:**
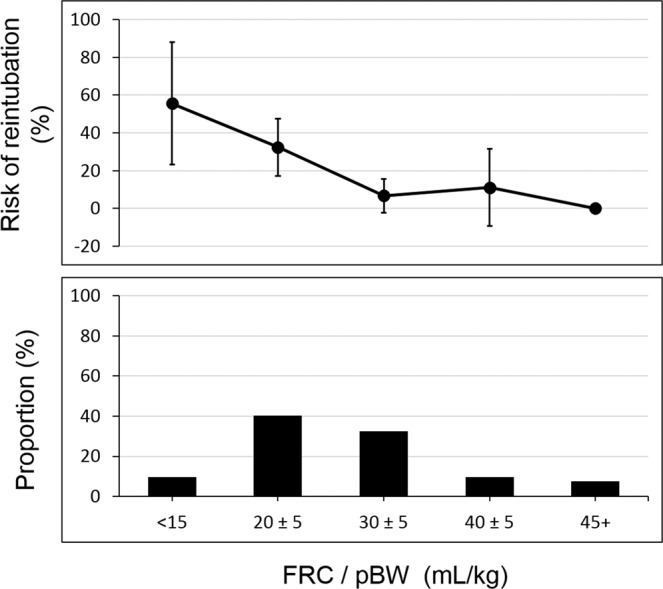
Distribution of functional residual capacity divided by predicted body weight (FRC/pBW) and probabilities of extubation success for every strata of FRC/pBW. Black bars indicate the proportion of patients and black lines with error bars indicate probabilities of extubation success with 95% confidence intervals.

Logistic regression analysis showed that reduced FRC was associated with higher risk of extubation failure (odds ratio, 1.14 per each decreased of 1 mL/Kg of FRC/pBW, 95% CI, 1.05–1.23, p = 0.002). Figure 2 shows the distribution of FRC/pBW and the probabilities of extubation success for every stratum of FRC/pBW. Reduced FRC/pBW was associated with lower probability of extubation success (p for trend = 0.002).

### Discrimination capacity of FRC

**Figure 3:**
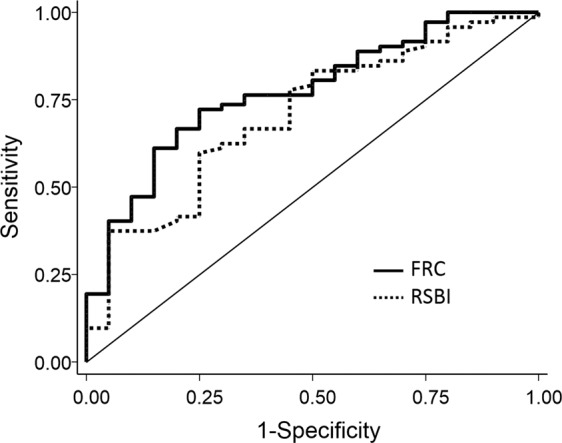
Area under the receiver operating characteristic curves (AUROCs) for discriminatory capacity for extubation success for functional residual capacity divided by predicted body weight (FRC/pBW) and rapid shallow breathing index (RSBI). The solid and dotted lines denote FRC and RSBI, respectively. AUROCs for FRC/pBW and RSBI were 0.77 (95% CI, 0.66–0.88) and 0.70 (95% CI, 0.58–0.83).

Figure 3 shows ROC curves of FRC/pBW and RSBI for discrimination of extubation success. The AUROCs were 0.77 (95% CI, 0.66–0.88) for FRC/pBW and 0.70 (95% CI, 0.58–0.83) for RSBI. The difference in AUROCs between FRC/pBW and RSBI did not reach statistical significance (p = 0.39).

Table 2 summarizes the diagnostic performance of FRC/pBW in predicting extubation outcome at different cut-off points. In the setting of using SBTs as the standard screening tool to assess readiness to extubate, the role of FRC/pBW measurement was to identify the patients at higher risk for extubation failure.

**Table 2:** Cut-offs of functional residual capacity divided by predictive body weight (FRC/pBW) in differentiating between extubation success and failure.

Cut-offs of FRC/pBW (mL/kg)	Sensitivity	Specificity	Positive predictive value	Negative predictive value
15	25%	94%	54%	82%
20	45%	82%	41%	84%
25	85%	60%	37%	84%
30	95%	38%	30%	96%
35	95%	21%	25%	94%

### FRC versus PaO_2_/FiO_2_ and tidal volume

**Figure 4:**
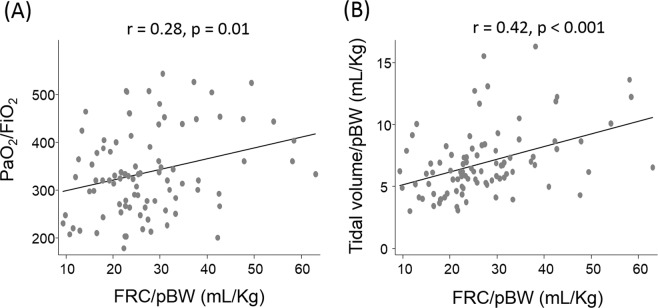
Scatters plots with regression lines for functional residual capacity (FRC) versus PaO_2_/FiO_2_ and FRC versus tidal volume of spontaneous breathing. pBW, predicted body weight. The analysis explored the effects of FRC reduction on oxygenation and ventilation.

Figure 4 shows the correlation between FRC versus PaO_2_/FiO_2_ and FRC versus the tidal volume of spontaneous breathing. The analysis explored the effects of FRC reduction on oxygenation and ventilation. There was a significant correlation between FRC and PaO_2_/FiO_2_ (r = 0.28, p = 0.01) and a moderate correlation between FRC and tidal volume of unassisted breathing (r = 0.42, p < 0.001).

## Discussion

This study showed that pre-extubation FRC was significantly associated with the risk of extubation failure in patients intubated for hypoxemic respiratory failure. FRC had acceptable discrimination capacity to predict extubation outcome with an AUROC of 0.77. Since FRC can now be reliably measured at the bedside in the ICU, this measurement may be used as a risk stratification tool to identify patients at higher risk for extubation failure owing to impaired alveolar aeration in the setting of using SBTs as the standard screening tool to assess readiness to extubate. The risk of extubation failure after passing an SBT is estimated to be 10–20%^14^, and reintubation is associated with poor outcome^15,16^. Clinical practice guidelines recommend extubating patients at high risk for extubation failure to preventative NIV^17^. The traditional risk factors for extubation failure include old age, coexisting COPD or congestive heart failure, and hypercapnia during the SBT^17^. Our study findings suggested that reduced FRC may be a new risk factor for extubation failure apart from the traditional risk factors.

We compared FRC and RSBI in their discrimination capacity for extubation failure in this study. The results suggested that FRC had similar discrimination capacity with RSBI in our study population. RSBI is a widely used predictor for extubation outcomes and is designed to evaluate a patient’s tolerance to spontaneous breathing^18,19^. A high RSBI score indicates a rapid shallow breathing pattern, which is an early sign of decompensation. In contrast, the measure of FRC is used to assess the change of lung volume in a disease status^2^. FRC usually decreases in diseases leading hypoxemic respiratory failure and increases in obstructive airway diseases. In our study, we tested the association between FRC and extubation outcomes in hypoxemic respiratory failure. Patients with hypercapnic respiratory failure were excluded because their change in FRC may be pathophysiologically different from that in patients with hypoxemic respiratory failure. Our findings suggested that reduced FRC was a risk factor for extubation failure. In the ICUs where bedside measurement of FRC is available, clinicians could consider using this modality to identify patients with increased risk for extubation failure, especially for patients with diseases that loss of lung volume is a key feature. However, this observational study did not test whether FRC can be used to guide the decision of extubation.

In this study, we used the ratio of FRC/pBW to evaluate the change in FRC in the individual patient. We think this is a practical approach to assess the change in FRC at the bedside, especially when reference values of FRC are not available. The range of FRC in a healthy individual measured in a sitting or standing position is around 30–35 mL/kg^6,7^. The FRC in our study (median, 25 mL/Kg, IQR, 20–32 mL/Kg) was below the normal reference values. The reduction of FRC observed in this study may be attributable to disease- and measurement-related factors. Diseases causing hypoxemic respiratory failure could result in a certain extent of loss of alveolar volume. Although the patients had been recovering from hypoxemic respiratory failure when the FRC was measured, the disease-related alveolar space loss may not be totally restored. The measured values of FRC are also affected by the settings of measurement. Previous studies have shown that FRC varies largely with sex, body height, age, posture, intubation, PEEP levels^8,20,21^. The European Respiratory Society provides an estimate of the FRC in healthy sitting individuals according to sex, height, and age^21^. In addition, FRC may decrease by 25% in healthy subjects in the supine position compared with the sitting position^8^. Intubated patients without pulmonary diseases could have 30–40% decreased FRC compared with predicted values^20^. The effect is probably related to a cranial shift of the diaphragm and loss of muscle tension among intubated patients.

We also evaluated the correlation between FRC and PaO_2_/FiO_2_ and the tidal volume of spontaneous breathing (Fig. 4). Our data showed that there was a significant correlation between FRC and PaO_2_/FiO_2_ and a moderate correlation between FRC and tidal volume of unassisted breathing. Similar findings have been observed in previous studies^22,23^. These findings support the idea that patients with reduced FRC before extubation have worse gas exchange and alveolar ventilation. To diminish the detrimental effects of FRC reduction, several modalities may be applied to reduce the risk of extubation failure. NIV provides inspiratory pressure support and PEEP, which may prevent further decruitment of injured lungs after extubation^24^. Prophylactic NIV support after extubation could be a reasonable intervention for this group of patients. High flow oxygen therapy is another feasible intervention after extubation because it can provide a low level of PEEP^25^.

The extubation failure rate in the current study is higher than the usual extubation failure rate of 10–15% in the study hospital. The possible reason accounted for the discrepancy may be related to less proportion of patients with simple weaning being included in the study. According to the study enrollment procedures, the patients with simple weaning were less likely enrolled in this study because the narrow time window between starting SBT and extubation. The data support this speculation are that the patients included in the current study had average two sessions of SBTs before the planned extubation. The outcome event rate may influence the distribution of FRC of the study cohort because higher extubation failure rates usually suggest poor respiratory reserve or lower FRC. In addition, a high event rate has substantial effect on positive predicted value and negative predicted value but have no effect on sensitivity and specificity.

Our study has certain limitations. First, this was a single center study in an Asian population. Multicenter studies are required to test the external validity of our study findings. Second, the majority of the study patients experienced pneumonia-induced hypoxemic respiratory failure; thus, it remains uncertain whether the study findings can be extrapolated to other types of disease related hypoxemic respiratory failure. Finally, we didn’t evaluate the cause of extubation failure. Reintubation due to upper airway problems is a potential confounder for our analysis. However, we had largely excluded the patients with potential upper airway problems by a cuff leak test and cough power evaluation.

## Conclusions

This prospective observational study demonstrated that pre-extubation FRC measured by a commercial bedside tool based on a nitrogen multiple breath washout technique had good discrimination capacity for extubation outcome. Bedside FRC measurement appears to be a practical tool that can be used to individualize the reintubation risk for patients with hypoxemic respiratory failure.

## Data Availability

The datasets used and analysed during the current study are available from the corresponding author on reasonable request.
